# Index Digit Necrosis as a Complication of Radial Artery Cannulation

**DOI:** 10.7759/cureus.28469

**Published:** 2022-08-27

**Authors:** Hesham R Alokaili, Tanveer A Bhat, Tareg M Alhablany, Tuqa A Alsinan, Duaa N Almansour, Felwa A AlMarshad, Abdulla Altamimi, Mohamed Ouhlous, Jawad Alnaqaa

**Affiliations:** 1 Department of Plastic and Reconstructive Surgery, King Saud Medical City, Riyadh, SAU; 2 Plastic and Reconstructive Surgery Section, Departement of Surgery, King Faisal Specialist Hospital and Research Centre, Riyadh, SAU; 3 Department of Radiology, King Saud Medical City, Riyadh, SAU; 4 Department of Plastic and Reconstructive Surgery, Al Kharj Military Industries Corporation Hospital, Al Kharj, SAU

**Keywords:** complication, hand ischemia, digit necrosis, radial artery, arterial cannulation

## Abstract

Arterial access is therapeutically and diagnostically useful. Its clinical utility is vast, and associated complications are infrequent. However, some unfortunate patients progress to disastrous outcomes. Luckily, ischemic hand complications are rare. Hand ischemia threatens independence and quality of life, thus warranting vigilance. We present a case of index digit necrosis as a complication of arterial cannulation in a 30-year-old patient with end-stage renal disease admitted to an intensive care unit.

## Introduction

Catheter-related hand ischemia is a rare complication due to the generous vascularity of the hand. This is evident by the wide difference in incidence between catheter-related radial artery occlusion and hand ischemia. The incidence of occlusion has been reported to be 25%, while hand ischemia is reported to be between 0.1% and 0.2% [1-3]. Furthermore, the predicted incidence of radial artery occlusion is likely under-reported due to it often being asymptomatic [1,4]. Some believe anatomic variance such as an incomplete superficial palmar arch increases the risk of this complication. However, angiographic studies of radially catheterized patients show preserved digital perfusion during radial occlusion, regardless of arch completeness [5]. Occlusion is inconsequential in most cases, with some bold clinicians catheterizing the ipsilateral ulnar artery in the presence of radial artery occlusion or even reusing an occluded radial artery [6-8].

Conventionally, the collateral circulation is tested prior to catheterization with Allen’s test; however, this appears to be out of tradition rather than having a concrete predictive power [9]. Although Allen’s test displays some correlation with thumb blood flow and lactate levels, its ability to predict ischemic complications is controversial [9,10]. The Barbeau test is an objective alternative relying on oximetry and plethysmography [9]. A study of 7000 trans-radially catheterized cardiac patients used Barbeau type D as an exclusion criterion with no reported hand ischemia [9]. In addition, even some patients who had a discouraging Allen's test underwent safe catheterization without ischemic hand complications [9]. The underlying process producing ischemia is thought to be a combination of thromboembolic phenomena and vasospasm arising from the inherent trauma of arterial cannulation.

## Case presentation

The patient is a 30-year-old male non-smoker who denies having any medical history prior to presentation. His COVID vaccination status is unknown. He was admitted with an acute presentation of end-stage renal disease. At presentation, he complained of coughing, shortness of breath, fatigue, vomiting, and generalized swelling for one week. On admission, the patient had tachycardia, tachypnea, and hypoxemia with maintained blood pressure and core body temperature. Admission to the intensive care unit followed. Treatment during early admission included mechanical ventilation, broad-spectrum antibiotics, serial right radial arterial blood gas sampling, and dialysis through the right internal jugular vein.

The patient’s admission was complicated by traumatic urinary catheterization, hypertension, and anemia requiring intermittent blood transfusions. In addition to these complications, the patient developed irreversible progressive critical ischemia of the right index finger within four days of admission. The patient was interviewed, and he denied having any previous medical problems, hospitalizations, operations, or antecedent trauma. He also denied the use of any prescription medicine, tobacco, illicit drugs, or alcohol. The patient denied a history of episodic spontaneous digit paresthesia or blue discoloration, which practically excludes Raynaud's phenomenon. The patient reported having healthy first-degree relatives living without any chronic or hereditary diseases. He was questioned with emphasis on hypercoagulable disorders such as hereditary thrombophilias or autoimmune disease, and he denied any family history. The presence of headaches, eye symptoms, malar rashes, generalized purpura, epistaxis, angina, hemoptysis, weakness, paresthesia in the extremities, hematuria, or any other sign of vasculitis is ruled out by a review of all systems.

**Figure 1:**
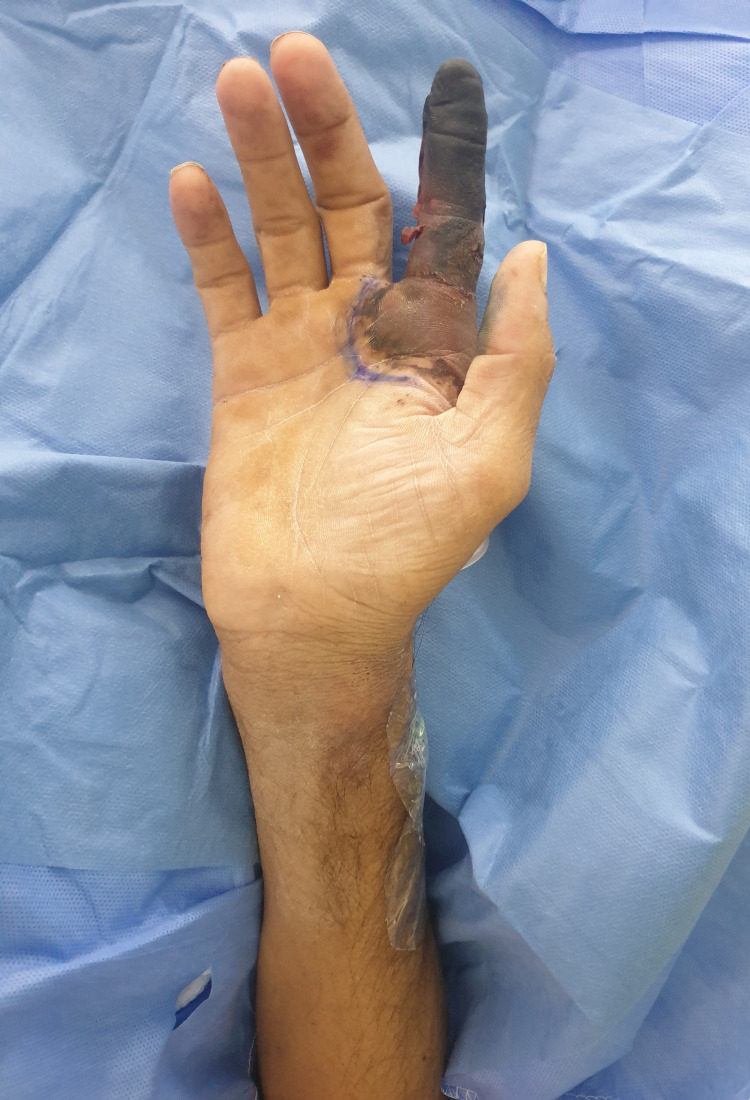
Volar view of the right upper extremity On examination, all digits were pink and viable except a dark, dry, and gangrenous right index digit. Gangrenous changes extended from the index’s fingertip to the middle interphalangeal crease. Pregangrenous changes were below the middle interphalangeal crease up to the first and second webspace reaching the proximal palmar crease and second metacarpal head from volar and dorsal, respectively. Ecchymosis and puncture marks over the snuff box due to arterial cannulation.

**Figure 2:**
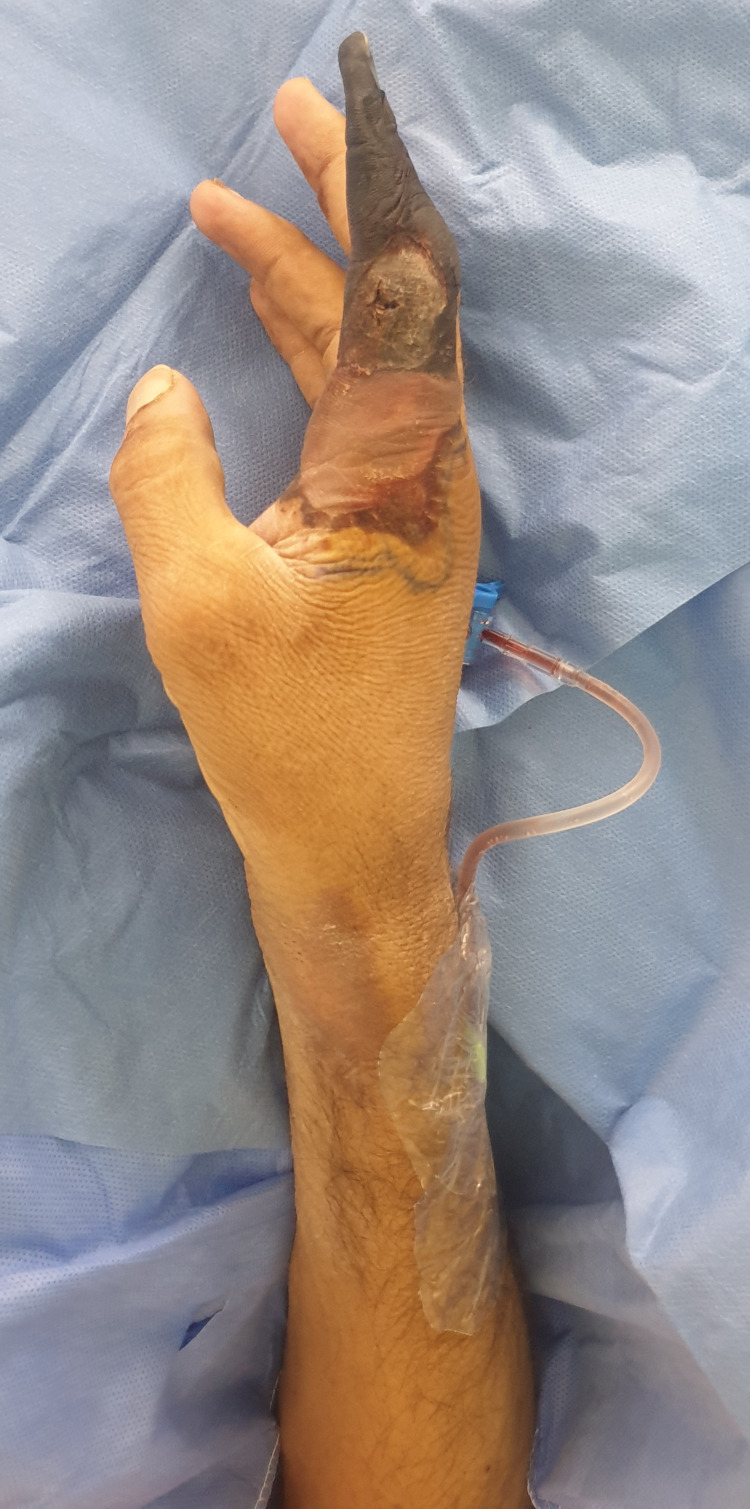
Lateral view right upper extremity Redemonstration of gangrenous changes from a lateral view. Redemonstration of ecchymosis at the snuffbox.

**Figure 3:**
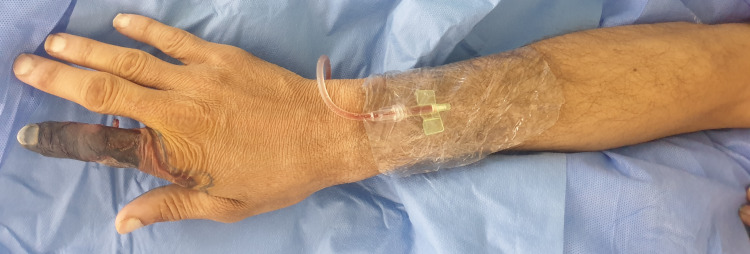
Dorsal view right upper extremity Redemonstration of gangrenous changes from a dorsal view. Redemonstration of ecchymosis at the snuffbox.

**Figure 4:**
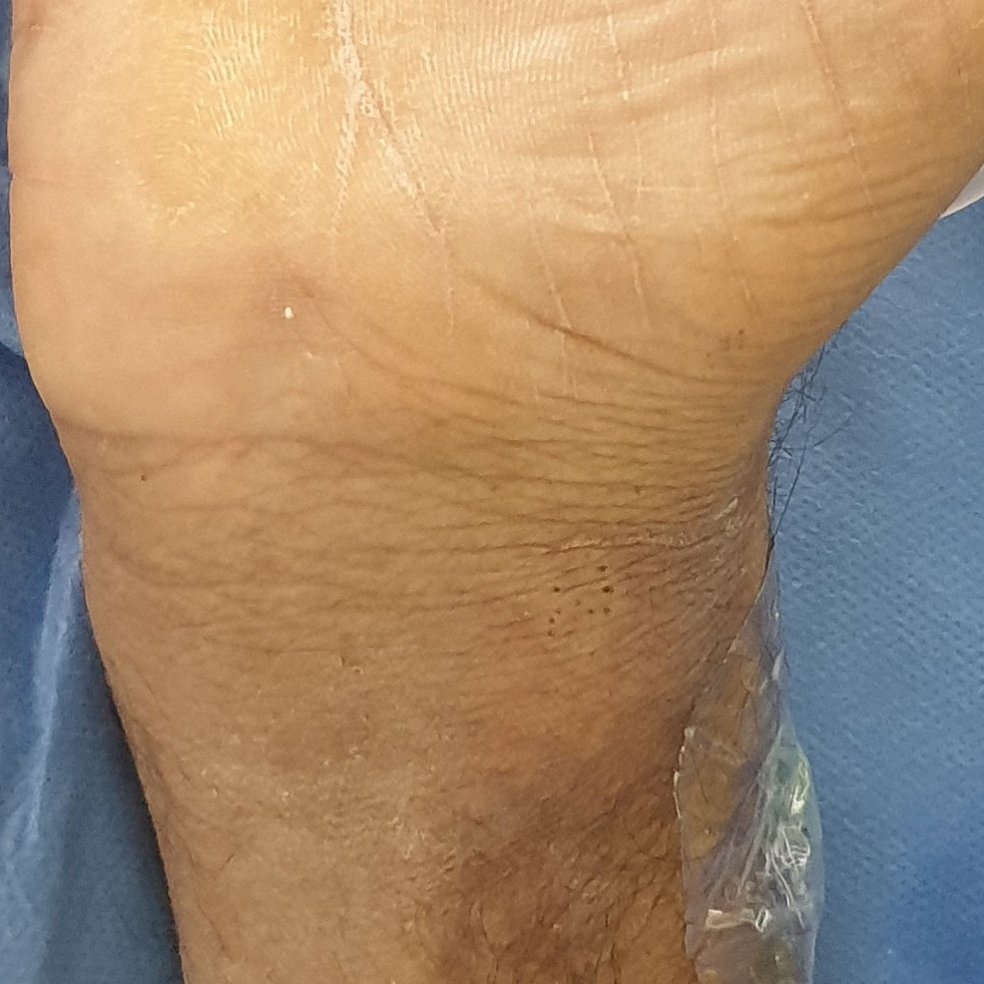
Magnified view of the right upper extremity Demonstration of ecchymosis and puncture marks over the snuff box due to arterial cannulation.

On examination, all digits were pink and viable except for a dark, dry, and gangrenous right index digit (Figures 1-3). Gangrenous changes extended from the index fingertip to the middle interphalangeal crease (Figures 1-3). Pregangrenous changes were below the middle interphalangeal crease up to the first and second webspace, reaching the proximal palmar crease and second metacarpal head from volar and dorsal, respectively (Figures 1-3). A small surrounding pre-gangrenous rim was present and regressed over the following days. The blue line marks the maximal progression of pregangrenous changes (Figures 1-3). All digits except the right index digit had normal neurovascular status with brisk capillary refill and preserved fine touch. Bilaterally, radial and ulnar pulses were palpable. Ecchymosis and multiple scattered puncture marks were observed proximal to the wrist crease over the snuff-box, and it was attributed to prior arterial cannulation (Figures 1-4). Chart reviewal showed maintenance of normal temperature and normal to high blood pressure throughout his admission without the usage of vasopressors. There was no evidence of sepsis or trauma. Usage of epinephrine mixed anesthetic or Allen’s test prior to cannulation was not documented.

Serial laboratory tests comprised complete blood counts, coagulation profiles, and basic chemistry panels. Most of the tests consistently displayed anemia and uremia. Platelet counts were low. Regardless of low platelets, there was no evidence of acquired thrombophilia disorders related to low platelets, such as hemolytic uremic syndrome, consumptive coagulopathy, and others. However, the patient is prone to thrombosis by virtue of his general condition and not of distinct clinical entities. Bed rest, edema, and renal disease all support thrombosis. Chest radiographs were consistent with fluid overload from renal failure. The right-hand radiograph did not show soft tissue opacification, cortical destruction, or other features of infection. Development of hemoserous bullae followed days after gangrenous changes, and a fluid culture was analyzed from the bullae aspirate. Blood and fluid cultures displayed no growth at five days.

**Figure 5:**
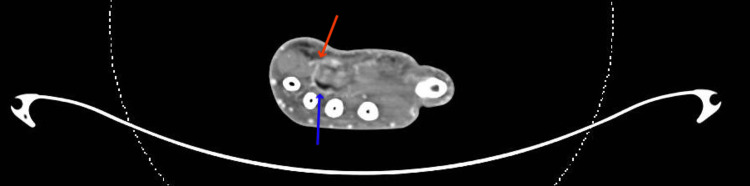
CT angiography The patency of the superficial palmar arch and the deep palmar arch are displayed. The red arrow indicates the superficial arch, while the blue arrow indicates the deep arch.

**Figure 6:**
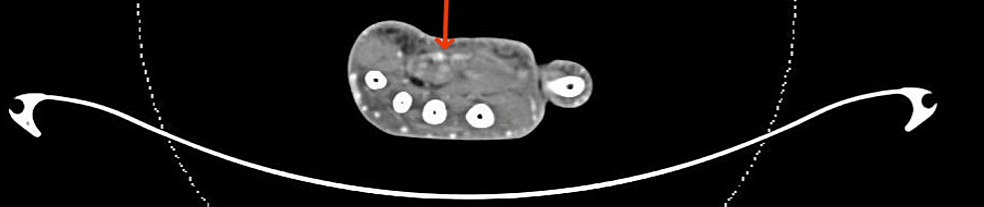
CT angiography The patency of the superficial palmar arch is redemonstrated in a more distal image. The superficial palmar arch is indicated by the red arrow.

CT angiography showed patent axillary, brachial, radial, and ulnar arteries. The patency of the superficial palmar arch and the deep palmar arch is demonstrated (Figure 4). The patency of the superficial palmar arch is redemonstrated in a more distal image (Figure 5). Further distal, the patency of the proper digital arteries in the little, ring, and middle fingers is demonstrated while demonstrating obliteration of the proper digital arteries of the index finger (Figure 6). Renal ultrasound showed small kidneys bilaterally with increased echogenicity that is consistent with end-stage renal disease. Definitive diagnosis by renal biopsy was considered contraindicated in view of the patient’s anemia and hemostatic impairment.

**Figure 7:**
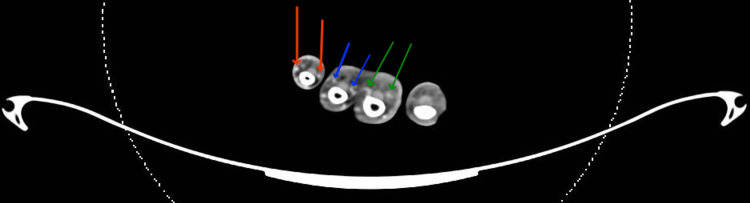
CT angiography The patency of the proper digital arteries in the little, ring, and middle fingers, while demonstrating obliteration of the proper digital arteries of the index finger. Indicators of the proper digital arteries for the little, ring, and middle fingers are red, blue, and green arrows, respectively.

The ischemia was irreversible, and the digit was left to autoamputate as the patient was reluctant to accept surgical intervention. Re-examination over the following days, displayed demarcation of necrosis, continued "mummification" of the digit, and preserved the neurovascular status of neighboring digits. The patient’s general condition had improved with dialysis, and the gangrene had not progressed further. Ultimately, the patient was discharged and lost to follow-up.

## Discussion

We believe that a constellation of physiological derangements predisposed the patient to develop digit necrosis. First, fluid overload and pulmonary congestion impaired oxygenation. Second, the patient’s anemia reduced oxygen delivery to the tissues. Third, the patient’s renal disease and uremia disrupted physiological hemostasis and created an environment that confusingly favors both hemorrhage and thrombosis [11]. Although the hemostatic condition of renal patients is beyond the scope of this paper, we briefly mention some mechanisms while referencing other more detailed papers.

Factors favoring hypercoagulability include a decreased activity of proteins C and S while having increased fibrinogen and von Willebrand factor [12]. In contrast, mechanisms favoring hemorrhage are related to imbalanced platelet activators, inhibitors, and the accumulation of toxins. Activators such as adenosine diphosphate and serotonin are reduced within platelets [13,14]. Some inhibitors, such as prostacyclin I2 and nitric oxide, are increased within platelets and plasma, respectively [15,16]. Toxins such as guanidinosuccinic acid accumulate in renal patients leading to impairment of adenosine diphosphate-related platelet aggregation [17]. Finally, platelets may be activated and consumed by interaction with dialysis membranes or through immunologic mechanisms such as heparin-induced thrombocytopenia [18,19].

Fourth, the patient’s generalized edematous presentation was contributed to by increasing diffusion distance between capillaries and cells, thus possibly producing relative tissue hypoxia [20]. Fifth, sampling of the right radial artery was likely a primary factor, as we shall discuss. The ischemia occurred acutely following arterial sampling within days of multiple punctures. Thus, the temporal relationship is suggestive of catheterization being a factor. The absence of neurovascular phenomena in all other digits points toward a local etiology, and thus the spatial relationship supports catheterization as a factor. In addition, the affected digit is predominantly supplied by the catheterized artery. Furthermore, the course between the radial artery and the index digit is semilinear. The linearity may support embolus transmission from the punctured radial artery to the index digit.

As stated, the generous vascularity of the hand is protective against ischemia even in the presence of radial artery occlusion. We speculate that an additional vascular pathology is needed to overcome collateralization in most cases. As stated, we derive this inference from differing incidences of radial occlusion and hand ischemia. The pathology may be in the form of vasospasm or thromboembolic processes in collateral circulation. Vasospasm of the radial artery may accompany thrombosis, and the incidence was estimated between 5% and 10% [21]. Interestingly, our case occurred in the absence of radial artery occlusion or vasospasm. Occlusion of the index’s proper digital arteries is suggestive of a thromboembolic process either developing in situ or transmitted from the radial artery (Figures 7). Cannulation predisposes thrombus formation by intimal damage. Additionally, high blood pressure may also have contributed to intimal damage by increasing shearing forces between the blood, cannula, and vessel wall. Finally, multiple punctures increased the likelihood of this complication (Figure 4) [21]. Patient-related risk factors such as smoking, diabetes mellitus, peripheral vascular disease, end-stage renal disease, and the Raynaud phenomenon have been referenced to coincide with radial thrombosis and post-cannulation ischemic complications [2,22]. Procedure-related risk factors have also been proposed, such as a low artery-to-sheath ratio and repeated arterial puncture [21].

As for treatment, we briefly display and direct toward published medical and operative interventions [2,22]. Medical management involves the use of vasodilators and anticoagulation [2]. The usage of nitroglycerin ointment, prilocaine, phentolamine, verapamil, diltiazem, heparin, and low molecular weight heparin has all been reported with varying efficacy [2,22-25]. No reported use of siloxanes was found during our review. As for surgical and endovascular interventions, many techniques have been described with varying outcomes, including catheter-directed thrombolysis, vein graft interposition, thrombectomy and angioplasty, embolus aspiration, venous arterialization, and chemical sympathectomy [2,22,26-31]. Progression to gangrene may occur regardless of technical success in revascularization [2].

## Conclusions

In conclusion, we recommend the judicious use of arterial catheters in patients predisposed to catheter-related ischemic complications. Risk factors for catheter-related ischemia include diabetes, renal disease, peripheral vascular disease, smoking, and Raynaud's phenomenon. We also recommend reducing procedure-related risk factors by minimizing arterial punctures and using a catheter size to achieve a high artery-to-sheath ratio. This report serves as an addition to the collection of literature on catheter-related hand ischemia. It also supports renal disease as being a risk factor for this complication. Regarding management, medical and surgical options can be considered according to the clinical situation.
